# Streptococcus agalactiae Meningoencephalitis Revealing Spondylodiscitis in a Diabetic Patient

**DOI:** 10.7759/cureus.90753

**Published:** 2025-08-22

**Authors:** Yousra Boughalem, Doha El Bekkaoui, Youssef El Kamouni, Lamiae Arsalane, Said Zouhair

**Affiliations:** 1 Department of Microbiology, Avicenna Military Hospital, Marrakesh, MAR; 2 Faculty of Medicine and Pharmacy of Marrakech, Cadi Ayyad University, Marrakesh, MAR; 3 Faculty of Medicine and Pharmacy of Agadir, Ibn Zohr University, Agadir, MAR

**Keywords:** bacterial meningoencephalitis, diabete mellitus, group b streptococcus, infectious spondylodiscitis, meningitis, streptococcus agalactiae

## Abstract

*Streptococcus agalactiae* is a well-known pathogen responsible for neonatal and postpartum infections. An increase in the incidence of invasive *Streptococcus agalactiae* infections has been observed in non-pregnant adults. Involvement of the central nervous system in adults remains rare, and its association with spondylodiscitis is exceptional. We report the case of a 65-year-old diabetic patient who presented with acute meningoencephalitis. Microbiological analysis of cerebrospinal fluid and blood cultures identified *Streptococcus agalactiae*. Despite initial clinical improvement, the patient developed paraparesis and low back pain. Subsequent lumbar imaging revealed L3-L4-L5 spondylodiscitis with epidural and soft tissue abscess. The spinal involvement was presumably caused by the same pathogen. This case highlights the importance of considering the involvement of *Streptococcus agalactiae* in adult central nervous system infections, especially in the presence of comorbidities.

## Introduction

*Streptococcus agalactiae*, also known as Group B *Streptococcus* (GBS), is commonly responsible for neonatal and postpartum invasive infections [1]. However, over the last two decades, the incidence of invasive *Streptococcus agalactiae* infections has significantly increased by two to fourfold among non-pregnant adults [2]. These infections can take a variety of clinical forms, although involvement of the central nervous system remains particularly uncommon [3]. In adults, *Streptococcus agalactiae* meningoencephalitis is a rare condition [4]. Similarly, spondylodiscitis due to *Streptococcus agalactiae* is also unusual [5], representing fewer than 2% of pyogenic spondylodiscitis [6]. The association of meningoencephalitis and spondylodiscitis due to *Streptococcus agalactiae* is exceptional, with only a few cases documented in the literature [7]. We report the case of a 65-year-old diabetic patient diagnosed with *Streptococcus agalactiae* meningoencephalitis, leading to the discovery of a spondylodiscitis with associated epidural and soft tissue abscess.

## Case presentation

A 65-year-old male patient, with no significant medical history aside from a left tibial fracture 37 years ago, complicated by chronic neuropathy treated with pregabalin for the last 14 months and a past pulmonary tuberculosis cured in 2020, was admitted to the emergency department with altered consciousness evolving over the past 48 hours in a febrile context.

The patient’s symptoms began approximately one week before admission, with the onset of a transient behavioral disorder characterized by psychomotor agitation, occurring in the context of unquantified fever. Due to the subsequent development of impaired consciousness, the patient was brought by his family to the emergency department.

Upon admission, the patient was febrile (38.6°C) and unconscious, with a Glasgow Coma Scale score of 11/15 [8]. Meningeal signs were present, including neck stiffness and positive Brudzinski and Kernig signs, without focal neurological deficits or seizure activity. Hemodynamically, the patient was normotensive (113/76 mmHg) but tachycardic (153 beats per minute) and polypneic (58 breaths per minute), with an oxygen saturation of 88% on room air. The remainder of the physical examination was unremarkable.

Given this clinical presentation, an infectious meningoencephalitis was suspected. Laboratory investigations (Table 1) revealed marked leukocytosis (28.6 × 10^3^/µL) with neutrophil predominance (91.7%), hyperglycemia (5.9 g/L), elevated C-reactive protein (320 mg/L), and procalcitonin (11.1 ng/mL). Hemostasis assessment and liver and renal function tests were within normal limits.

**Table 1:** Laboratory investigations results on admission.

Parameters	Values	Reference range
White blood cells (×10^3^/µL)	28.6	4–11
Neutrophils (×10^3^/µL)	26.25	1.4–7.7
Neutrophils (%)	91.7	40–70
Hemoglobin (g/dL)	14.3	13–18
Platelet count (×10^3^/µL)	256	150–450
Activated partial thromboplastin time (seconds)	27	26–42
Prothrombin time (%)	80.3	70–100
Glucose (g/L)	5.9	0.7–1.1
Creatinine (mg/L)	10.55	6.8–13.6
Urea (g/L)	0.22	0.15–0.45
Total bilirubin (µmol/L)	13	<17
Aspartate aminotransferase (U/L)	23	<50
Alanine aminotransferase (U/L)	12	<65
Alkaline phosphatase (U/L)	112	40–129
C-reactive protein (mg/L)	320	<5
Procalcitonin (ng/mL)	11.1	<0.5

A full infectious workup was initiated, including a lumbar puncture, blood cultures, a urine culture, which returned sterile the following day, and a chest radiography that was unremarkable. On the same day of admission, an empirical antibiotic therapy with intravenous ceftriaxone (3 g every 12 hours) was started, in combination with methylprednisolone (40 mg every six hours), and the patient was transferred to the intensive care unit for further management.

**Figure 1:**
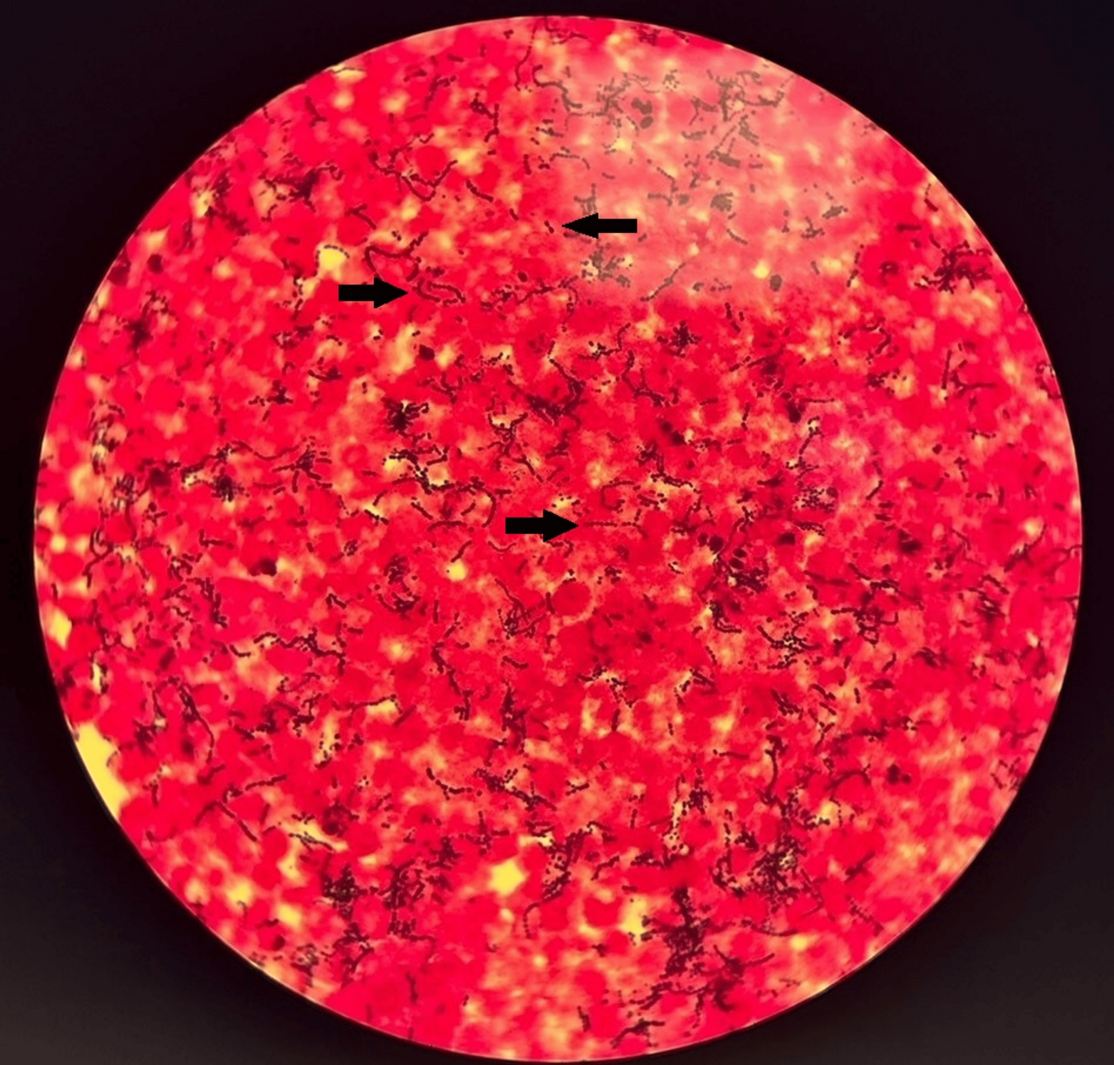
Cerebrospinal fluid Gram stain showing Gram-positive cocci in pairs and chains.

Cerebrospinal fluid (CSF) cytobacteriological examination revealed a purulent appearance with 44,000 leukocytes/mm³, 97% of which were neutrophils, and numerous Gram-positive cocci on Gram stain (Figure 1). CSF biochemistry showed elevated protein levels (11.5 g/L) and hypoglycorrhachia (1.93 g/L), below two-thirds of the concurrent serum glucose. Due to the suggestive findings, a multiplex polymerase chain reaction (FilmArray® Meningitis/Encephalitis (ME) Panel, BioMérieux) was performed, which identified *Streptococcus agalactiae* in the CSF. A sample of the CSF was then stored at -20°C in case it was required for additional molecular biology testing.

**Figure 2:**
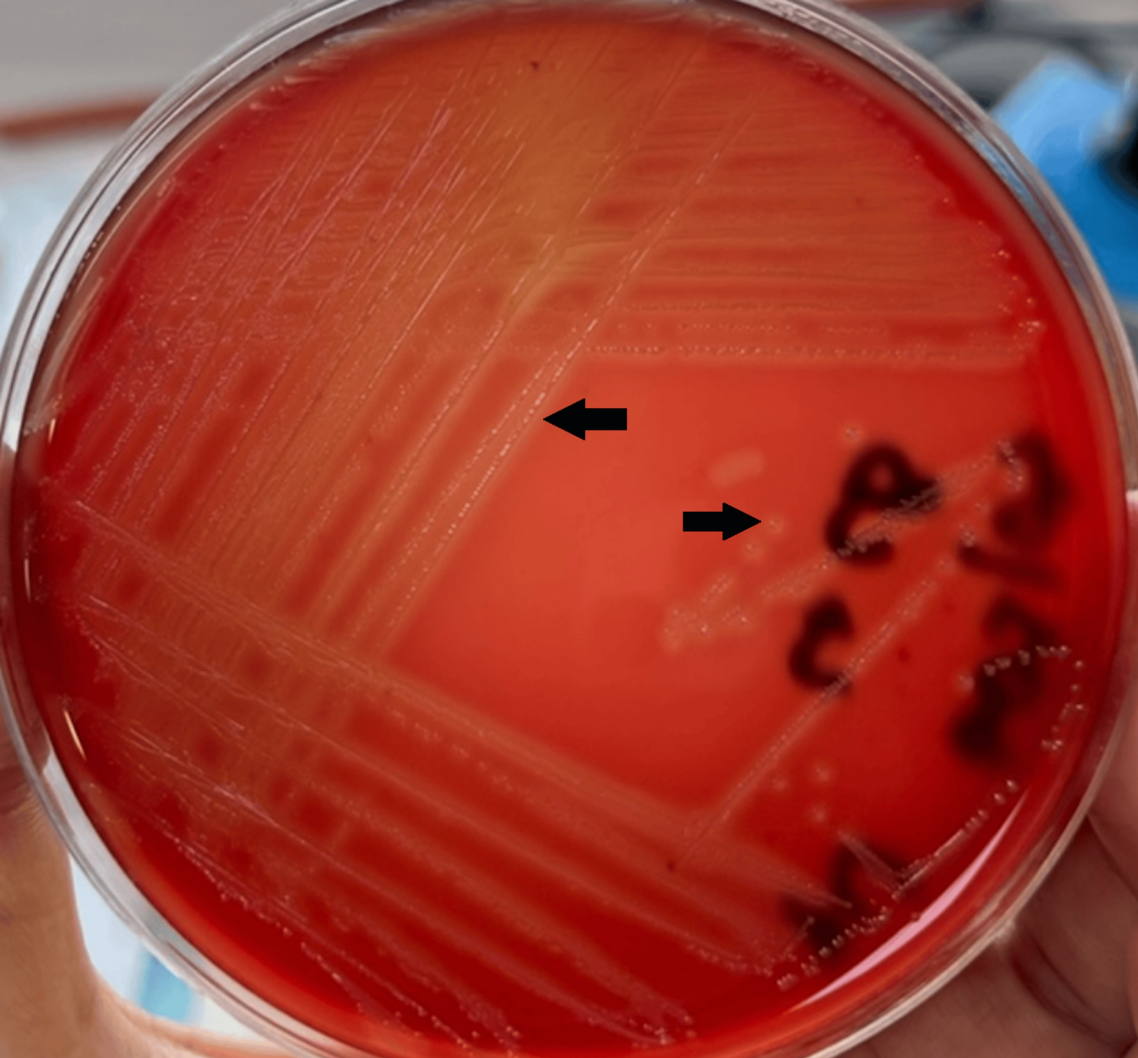
Streptococcus agalactiae colonies on blood agar. The arrows show grayish beta-hemolytic colonies of *Streptococcus agalactiae*.

After 24 hours of incubation, the blood agar culture of the CSF yielded small, grayish, beta-hemolytic colonies of Gram-positive cocci (Figure 2) identified as *Streptococcus agalactiae* using the BD Phoenix™ M50 automated system. Antimicrobial susceptibility testing, interpreted according to CASFM/EUCAST (Comité De l'Antibiogramme de la Société Française de Microbiologie/European Committee on Antimicrobial Susceptibility Testing) 2024 guidelines [9], showed susceptibility to ceftriaxone and isolated resistance to tetracycline (Table 2), thus confirming the appropriateness of the initiated antibiotic regimen.

**Table 2:** Antimicrobial susceptibility testing results. MIC : Minimal inhibitory concentration

Antibiotics	MIC (µg/mL)	Clinical categorization
Penicillin G	≤0.03	Susceptible
Amoxicillin	≤0.25	Susceptible
Ceftriaxone	≤0.5	Susceptible
Cefotaxime	≤0.5	Susceptible
Cefepime	≤0.5	Susceptible
Meropenem	≤0.13	Susceptible
Moxifloxacin	≤0.25	Susceptible
Vancomycin	≤0.5	Susceptible
Teicoplanin	≤1	Susceptible
Tetracycline	>4	Resistant
Erythromycin	≤0.06	Susceptible
Clindamycin	0.06	Susceptible
Linezolid	1	Susceptible

Concurrently, both blood cultures collected on admission and incubated in the BD BACTEC™ system returned positive after three days of incubation. Identification via the FilmArray® Blood Culture Identification 2 (BCID2) panel (BioMérieux) confirmed the presence of *Streptococcus agalactiae*, with an antibiogram matching that of the strain isolated from the CSF.

During hospitalization in the intensive care unit, a persistent hyperglycemia prompted the initiation of insulin therapy. HbA1c measurement revealed a value of 11.1%, consistent with a previously undiagnosed diabetes mellitus [10]. Concurrently, the patient showed improvement with resolution of fever, decrease in inflammatory markers, and gradual recovery of consciousness.

On the sixth day of hospitalization, the patient regained full consciousness but presented with retrograde amnesia, was unable to remember recent events surrounding his illness, and reported low back pain. Neurological examination revealed a motor deficit in both lower limbs with loss of deep tendon reflexes, muscular hypotonia of both lower limbs, bowel and bladder dysfunction, a positive Babinski sign, no coordination disorders, and the absence of radiculopathy with a negative Lasègue’s sign. These findings led to the suspicion of spondylodiscitis.

The patient was transferred the same day to the infectious diseases department for further etiological investigation. Upon admission to the department, the patient underwent a full assessment, including a transthoracic echocardiography that showed a normal heart function with no evidence of infectious endocarditis, routine laboratory investigations (Table 3), and HIV, Hepatitis B virus (HBV), and Hepatitis C virus (HCV) serologies. Viral serologies for HIV and HCV were negative, while serological markers for HBV indicated a resolved infection (negative HBsAg, and positive anti-HBs and total anti-HBc). Due to the strong clinical suspicion of spondylodiscitis and the patient’s history of tuberculosis, sputum and previously collected CSF were tested for *Mycobacterium tuberculosis* using the Xpert® MTB/RIF Ultra technology and showed negative results.

**Table 3:** Laboratory investigations results on the sixth day of hospitalization.

Parameters	Values	Reference range
White blood cells (×10^3^/µL)	18.5	4–11
Neutrophils (×10^3^/µL)	16	1.4–7.7
Neutrophils (%)	86.5	40–70
Hemoglobin (g/dL)	14.1	13–18
Platelet count (×10^3^/µL)	278	150–450
Activated partial thromboplastin time (seconds)	28	26–42
Prothrombin time (%)	81	70–100
Glucose (g/L)	2.3	0.7–1.1
Creatinine (mg/L)	9.5	6.8–13.6
Urea (g/L)	0.25	0.15–0.45
Total bilirubin (µmol/L)	12	<17
Aspartate aminotransferase (U/L)	24	<50
Alanine aminotransferase (U/L)	15	<65
Alkaline phosphatase (U/L)	110	40–129
C-reactive protein (mg/L)	138.4	<5
Procalcitonin (ng/mL)	5.6	<0.5

**Figure 3:**
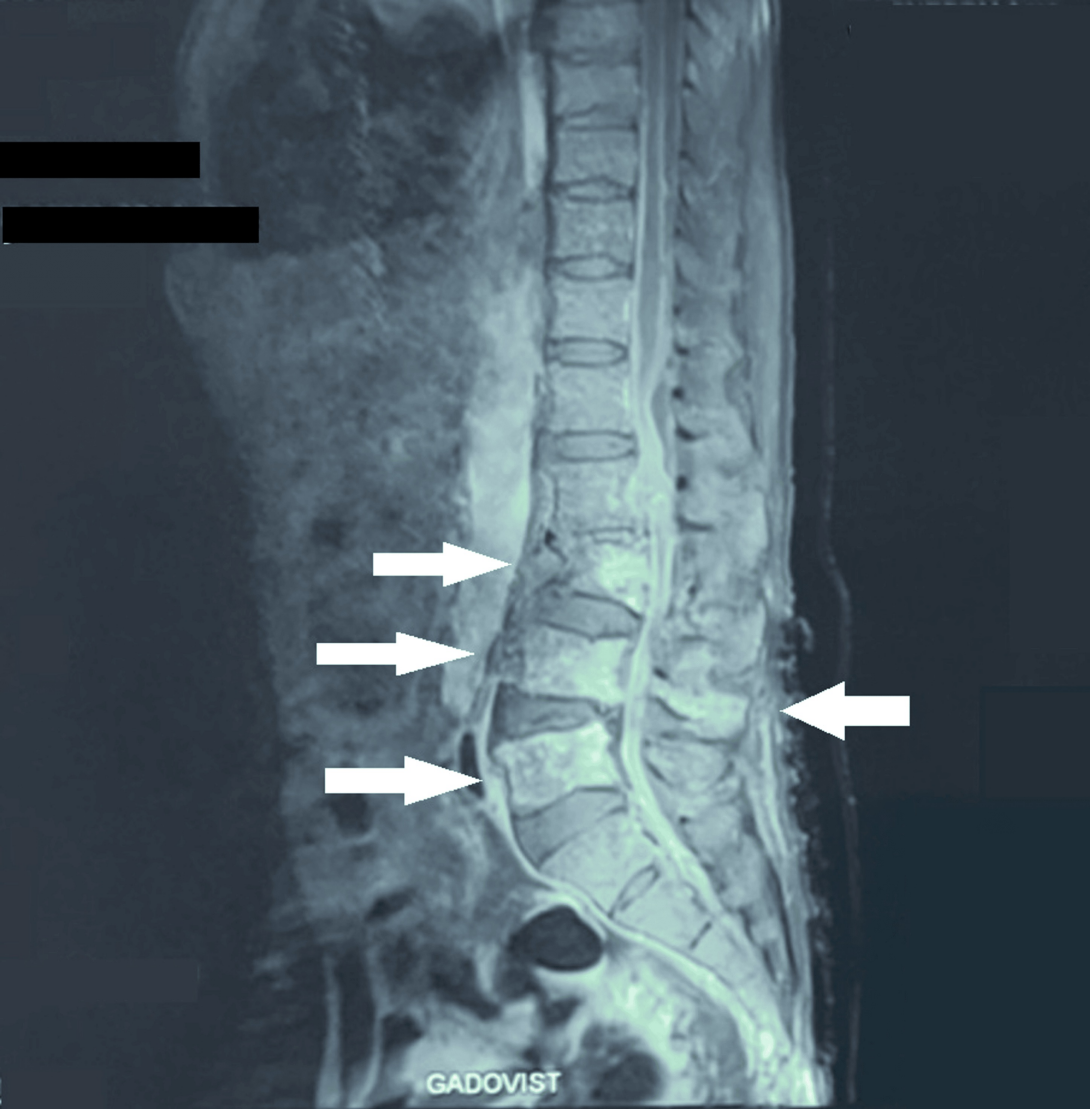
Sagittal section lumbar MRI after contrast injection showing L3-L4-L5 spondylodiscitis with adjacent epidural and soft tissue collections.

The following day, lumbar MRI revealed infectious L3-L4-L5 spondylodiscitis with adjacent epidural and soft tissue collections (Figure 3). Surgical management was planned. However, the patient suddenly experienced chest pain, followed by severe bradycardia, resulting in cardiopulmonary arrest. Despite resuscitation efforts, the arrest was unrecoverable, and the patient died later that day.

## Discussion

*Streptococcus agalactiae* is a Gram-positive, catalase-negative cocci that usually colonizes the respiratory, gastrointestinal, and urogenital systems of healthy adults [2,4]. Although predominantly implicated in puerperal and neonatal infections [1], the occurrence of invasive *Streptococcus agalactiae* infections in non-pregnant adults has increased by two to fourfold over the last two decades, especially in elderly and immunocompromised subjects [2]. This was highlighted by two studies from the United States, which showed an increase in the incidence of invasive *Streptococcus agalactiae* infections among non-pregnant adults. In the studies, the number of cases increased from 3.6 per 100,000 persons in 1990 to 7.3 in 2007 [11], and from 8.1 in 2008 to 10.9 in 2016 [12].

In non-pregnant adults, the clinical manifestations associated with invasive *Streptococcus agalactiae* infections include bacteremia, skin, soft tissue, and osteoarticular infections, urosepsis, pneumonia, peritonitis, endocarditis, and meningitis [13]. Because of its inability to cross the blood-brain barrier easily in adults [2], meningitis caused by *Streptococcus agalactiae* remains exceptional, accounting for only 2% of these invasive infections [14]. Moreover, *Streptococcus agalactiae* has been reported to be responsible for only 0.3% to 4.3% of the cases of bacterial meningitis among non-pregnant adults [15]. The clinical presentation of *Streptococcus agalactiae* meningitis is non-specific, and CSF findings are similar to those seen in other cases of bacterial meningitis [3], typically showing neutrophilic pleocytosis, elevated protein levels, and low glucose levels [16]. Definitive diagnosis relies on microbiological testing [3]. However, it has been reported that *Streptococcus agalactiae* meningitis may present earlier due to the early onset of encephalopathic symptoms [17,18]. When compared to other causes of bacterial meningitis, *Streptococcus agalactiae* meningitis is associated with higher rates of morbidity and mortality [3].

In Morocco, cases of *Streptococcus agalactiae* meningitis are still very rare. To our knowledge, only two cases of meningoencephalitis, including the present case, and one isolated case of meningitis have been reported in the literature [14,16]. This low number may be due to under-detection, late diagnosis, or a low actual incidence in our population.

Invasive infections caused by *Streptococcus agalactiae* are more likely to occur in individuals with certain risk factors. These include advanced age, diabetes mellitus, malignancies, chronic alcoholism, liver disease (such as cirrhosis), chronic renal failure, and immunosuppressive conditions or therapies [14]. Among these, diabetes and older age appear to be the most consistently reported independent risk factors in the literature [3]. A systematic review by van Kassel et al. analyzed 141 cases of *Streptococcus agalactiae* meningitis, finding a mean age of 56 years. Among these patients, 38% were immunocompromised, with diabetes mellitus being the most common underlying condition (20%) [7]. Our patient was 65 years old and had diabetes mellitus, two main susceptibility factors for developing *Streptococcus agalactiae* invasive infections.

*Streptococcus agalactiae* can lead to infection through direct inoculation, extension from nearby infected tissues, or, most commonly, via hematogenous dissemination [3]. In many cases, the exact origin of the infection remains unidentified [4]. Van Kassel et al. found a focus of infection outside the central nervous system in only 45% of the patients, with endocarditis (12%) and otitis/sinusitis (12%) being the principal sources of infection, followed by arthritis (9%), whereas spondylodiscitis was only described in four (3%) cases [7]. In our case, meningoencephalitis due to *Streptococcus agalactiae* led to the diagnosis of an infectious L3-L4-L5 spondylodiscitis, complicated by an adjacent epidural and soft tissue abscess. Although no microbiological confirmation from the spinal site was obtained, the vertebral involvement was most likely caused by *Streptococcus agalactiae*. Several elements support the possibility of a streptococcal cause of the spondylodiscitis. First, the chest radiography performed on admission was normal, and the GeneXpert tests performed on both sputum and CSF were negative. These findings made it reasonable to rule out pulmonary and neuromeningeal tuberculosis. In contrast, *Streptococcus agalactiae* was isolated simultaneously in CSF and blood cultures, which suggests hematogenous dissemination of the pathogen to the spinal site. In the absence of any other documented source of infection and given the chronological sequence of symptoms, with the meningeal syndrome preceding the development of back pain and paraparesis, spondylodiscitis likely represented a secondary focus of infection via hematogenous spread. However, the hypothesis that a primary spondylodiscitis extended contiguously to the meninges, causing the meningoencephalitis, is also plausible. In this scenario, the initial low back pain may have been masked by pregabalin use, as the patient was taking it before his admission [19]. This may have led to a clinical presentation dominated by acute meningeal symptoms that prompted hospital admission. In this case, the bacteriemia could have occurred as a result of the secondary meningeal infection.

The treatment of *Streptococcus agalactiae* meningitis typically involves intravenous third-generation cephalosporins, such as ceftriaxone, administered for approximately two weeks. However, if associated with spondylodiscitis, a longer course of antibiotic therapy may be required [4]. In our case, the patient received an empirical antibiotherapy consisting of high-dose ceftriaxone. The treatment was maintained after the antibiogram showed a multi-susceptible germ. An improvement was observed during the treatment, marked by apyrexia, full recovery of consciousness, and a decrease in infectious parameters. However, the patient’s condition was marked by the development of acute chest pain followed by severe bradycardia, rapidly progressing to cardiopulmonary arrest. Although no autopsy was conducted, the hypothesis of acute myocardial infarction is considered likely, particularly as the absence of abnormalities on transthoracic echocardiography does not rule it out.

The prognosis is often poor in patients with *Streptococcus agalactiae* meningitis, particularly in the elderly and those with comorbidities, with a high mortality rate frequently resulting from systemic complications such as cardiorespiratory failure, multiorgan dysfunction, or disseminated intravascular coagulation [3,14]. Van Kassel et al. reported a mortality rate of 31%. Death occurred as a result of respiratory or cardiac arrest in 13% of those patients and was related to advanced age and immunodeficiency [7]. In our case, the patient showed improvement under antibiotic treatment, but his age and underlying diabetes, within the context of an ongoing infectious and inflammatory state, likely contributed to the fatal outcome.

## Conclusions

This case reports a rare presentation of *Streptococcus agalactiae* meningoencephalitis in a 65-year-old, previously undiagnosed diabetic patient. The meningoencephalitis led to the diagnosis of an underlying L3-L4-L5 spondylodiscitis with adjacent epidural and soft tissue abscess. Despite the initial improvement of the patient, his condition deteriorated, resulting in a fatal outcome. This case highlights the importance of considering the involvement of *Streptococcus agalactiae* in adult central nervous system infections, especially in the presence of comorbidities such as diabetes mellitus.
